# Trophic ecology, habitat, and migratory behaviour of the viperfish *Chauliodus sloani* reveal a key mesopelagic player

**DOI:** 10.1038/s41598-020-77222-8

**Published:** 2020-12-02

**Authors:** Leandro Nolé Eduardo, Flávia Lucena-Frédou, Michael Maia Mincarone, Andrey Soares, François Le Loc’h, Thierry Frédou, Frédéric Ménard, Arnaud Bertrand

**Affiliations:** 1 Departamento de Pesca e Aquicultura, Universidade Federal Rural de Pernambuco, Recife, PE Brazil; 2 Institut de Recherche Pour le Développement (IRD), MARBEC, Univ. Montpellier, CNRS, Ifremer, IRD, Sète, France; 3 Instituto de Biodiversidade e Sustentabilidade, Universidade Federal do Rio de Janeiro, Caixa Postal 119331, Macaé, RJ 27910-970 Brazil; 4 IRD, Univ. Brest, CNRS, Ifremer, LEMAR, IUEM, 29280 Plouzane, France; 5 Aix Marseille Univ., Université de Toulon, CNRS, UM110 Marseille, IRD, MIO France; 6 Departamento de Oceanografia, Universidade Federal de Pernambuco, Recife, PE Brazil

**Keywords:** Ecology, Animal migration, Behavioural ecology, Ecosystem ecology, Ecosystem services, Population dynamics, Stable isotope analysis, Tropical ecology

## Abstract

Mesopelagic fishes are numerically the most important vertebrate group of all world’s oceans. While these species are increasingly threatened by anthropogenic activities, basic biological knowledge is still lacking. For instance, major uncertainties remain on the behaviour, ecology, and thus functional roles of mesopelagic micronektivores, particularly regarding their interactions with physicochemical features. Here, we examine the trophic ecology, habitat, and migratory behaviour of the viperfish (*Chauliodus sloani*)—a poorly known and abundant deep-sea species—to further understand the ecology and thus functional role of mesopelagic micronektivores. Moreover, we explore how physical drivers may affect these features and how these relationships are likely to change over large oceanic areas. The viperfish heavily preys on epipelagic migrant species, especially myctophids, and presents spatial and trophic ontogenetic shifts. Temperature restricts its vertical distribution. Therefore, its trophodynamics, migratory behaviour, and functional roles are expected to be modulated by the latitudinal change in temperature. For instance, in most tropical regions the viperfish stay full-time feeding, excreting, and serving as prey (e.g. for bathypelagic predators) at deep layers. On the contrary, in temperate regions, the viperfish ascend to superficial waters where they trophically interact with epipelagic predators and may release carbon where its remineralization is the greatest.

## Introduction

Mesopelagic fishes (200–1000 m depth) are numerically the most important vertebrate group of the world’s oceans^1^, usually presenting global distribution^2,3^, high biodiversity^4^, and several adaptations to overcome challenges imposed by the deep-sea^4^. Most of these species vertically migrate to the surface to feed at night and actively transport the ingested carbon to deep waters during daylight^5^, a pathway enhancing the oceanic carbon storage and thus global carbon cycles^6–8^. Moreover, they are an important trophic link for the maintenance of harvestable fish stocks^9–11^ and the connection between shallow and deep-sea ecosystems^12^. However, while there is a major lack of knowledge regarding their global composition, ecology, and ecosystem functions^13–15^, these species are increasingly threatened by anthropogenic activities. For instance, effects of climatic change^16,17^, plastic pollution^18^, and exploitation of deep-sea resources^15,19^ stand to alter the structure and function of deep-sea ecosystems. Therefore, as threats to the diversity and stability increase, the understanding of mesopelagic ecosystems, their processes, and functions is mandatory, especially when sustainability is intended to be achieved^15,20^.

Although research on mesopelagic species has considerably advanced over the past few years^3,5,20–24^, most works focused on zooplanktivorous groups (e.g. myctophids, sternoptychids), while less attention has been paid to micronektivores (e.g. stomiids) that occupy higher trophic levels^25^. Given their high abundance^26^, deep migrations^26,27^, great body mass^4^, and high predation on migrant zooplanktivorous fishes^23^, mesopelagic micronektivores are a crucial component of deep-sea systems that hitherto has been overlooked. Indeed, the trophic ecology, migratory behaviour, and environmental interactions of mesopelagic micronektivores remains poorly known worldwide and unexplored in most oceanic areas^13,15,25^. It is therefore not clear how physical drivers (e.g. temperature, oxygen) structure these communities and how these relationships are likely to change in the space and time. Additionally, most of the previous studies addressing the trophodynamics of micronektivores do not include their predators and/or were based solely on stomach contents^25,27,28^, while further approaches (e.g. stable isotopes, fatty acids, genetics) are required to provide a comprehensive picture of energy flows across trophic compartments^20^. Clarification of the ecology, vertical behaviour and trophic relationships of micronektivores should provide key knowledge on mesopelagic communities and systems^13–15^. Moreover, it may help to understand how these species might respond under climatic changes^16^ and what consequences it may have for their functional role and thus ecosystems health.

In this context, here we examine the habitat, trophic ecology, and vertical migration of the viperfish *Chauliodus sloani* (Stomiiformes: Stomiidae)—a poorly known and abundant deep-sea species^29,30^—to further understand the ecology and thus functional role of mesopelagic micronektivores. For that, we combine several approaches and take advantage of a multidisciplinary deep-sea survey around oceanic islands and seamounts in the western Tropical Atlantic. First, we assess the trophic ecology of the viperfish by coupling stomach content analyses with an extensive stable isotopic data (carbon and nitrogen) of its main probable trophic links, including zooplankton, crustaceans, fish larvae, zooplanktivorous fish, and epipelagic and bathypelagic potential predators. Second, we assess viperfish migratory behaviour by using novel information on its abundance, distribution, and physicochemical characteristics of its habitat (temperature and oxygen). Additionally, we combine our results with previous studies to construct a conceptual model, examining how temperature might influence trophic ecology and vertical movements of the viperfish and thus how latitudinal changes in sea temperature can affect its potential contribution to carbon sequestration.

## Materials and methods

### Specimens and data collection

**Figure 1:**
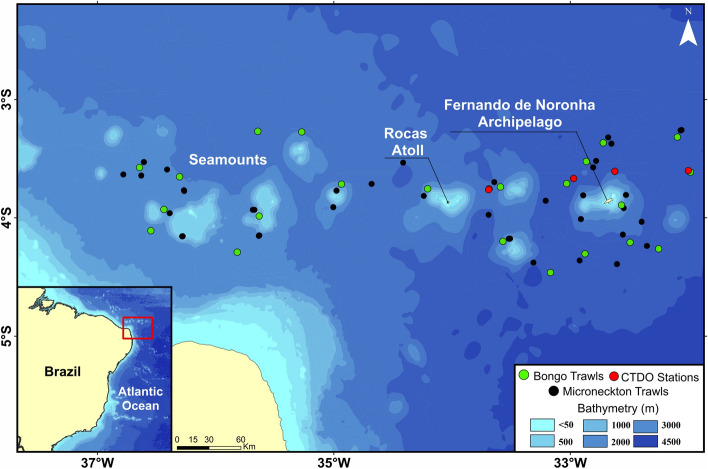
Study area (Fernando de Noronha Ridge) with CTD, bongo, and micronekton-trawl sampling stations. This map was created using the software Qgis 3.14 (https://www.qgis.org/pt_BR/site).

Specimens and data collection are described as follows in^20,31^. Data were collected off northeastern Brazil (Fernando de Noronha Ridge) during the Acoustics along the BRAzilian COaSt 2 (ABRACOS2) survey, carried out from 9th April to 6th May 2017, onboard the French RV *Antea*. Sampling of mesopelagic fishes, crustaceans and gelatinous organisms was conducted during day and night at 33 stations by using a micronekton trawl (body mesh: 40 mm, cod-end mesh: 10 mm) from 10 to 1,113 m (Fig. 1, Suppl. Material 1). Targeted depth was defined for each tow according to the presence of acoustic scattered layer or patches as observed using a Simrad EK60 (Kongsberg Simrad AS) split-beam scientific echosounder, operating at 38, 70, 120 and 200 kHz. Each trawl was performed for about 30 min at 2–3 kt. Tow duration was considered from the moment of the arrival of the net on the pre-set depth to the lift-off time, recorded utilizing a SCANMAR system. The net geometry has also been monitored using SCANMAR sensors, to give headline height, depth, and distance of wings and doors to ensure the net was fishing correctly. As the trawl was not fitted with opening or closing mechanism, the collection of specimens during the lowering or hoisting of the net was reduced as much as possible by decreasing ship velocity and increasing winch speed.

Temperature, salinity, oxygen, and fluorescence profiles were collected using a CTDO (model: SeaBird911 + ; Fig. 1). Particulate organic matter (POM) was sampled at 22 stations by filtering seawater from the maximum fluorescence depth (~ 80 m depth) through GF/F filters (47 mm), followed by a dry proceeding of 36 h (40 °C)^32^. Zooplankton samples were collected using bongo nets (four nets fitted with 64, 120, 300, and 500 µm mesh sizes) that were towed from 200 m depth up to the surface at 22 stations. Additional epipelagic sampling, targeting top predators, was performed aboard a sportfishing boat around the Fernando de Noronha Archipelago using hook and line.

Captured organisms were fixed in a 4% formalin solution for one month and then preserved in a 70% alcohol solution. At the laboratory, individuals were identified to the lowest taxonomic level, measured (nearest 0.1 cm of standard length, SL) and weighed (nearest 0.01 g of total weight, TW). Voucher specimens were deposited in the NPM – Fish Collection of the “Instituto de Biodiversidade e Sustentabilidade, Universidade Federal do Rio de Janeiro” (UFRJ). The authors confirm that all methods were approved and carried out in accordance with relevant guidelines and regulations of the Brazilian Ministry of Environment (SISBIO; authorization number: 47270–5).

### Vertical distribution, habitat, and migration

Viperfish vertical behaviour was characterised by using data on diel vertical distribution of abundance, size distribution, and physicochemical habitat. The relative index of abundance (Catch Per Unit of Effort—CPUE) was calculated considering the number and weight of specimens captured per hour, standardized to a similar net-mouth area of 120 m^2^ (estimated through SCANMAR sensors). These values, as well as the mean length and weight of specimens, were considered according to the diel period (day/night), and depth strata (10–1000 m, intervals of 100 m). Day was considered to extend from one hour after sunrise to one hour before sunset, while the night was from one hour after sunset to one hour before sunrise. Dawn or dusk samples were discarded when studying day/night vertical distributions. Except for the layers 200–300 and 700–800 at night, where no aggregation of organism was observed through acoustics, all depth strata were sampled at least once (Suppl. Material 1). A two-way ANOVA was performed^33^ to determine significant differences in SL and TW between period of the day and depth strata, following the necessary assumptions of normality (Kolmogorov–Smirnov test) and homoscedasticity (Levene’s test). Distribution pattern of specimens concerning their environment was analysed by combing data on vertical distributions and mean profiles of temperature and oxygen.

### Trophic ecology

Gut Content (GCA) and carbon and nitrogen Stable Isotopes Analyses (SIA) were implemented to assess the trophic ecology of the viperfish. Both analyses were performed considering three size classes (< 15 cm; > 15 cm; and pooled sizes), based on the viperfish size at sexual maturity (L_50_:15 cm)^34^. Additionally, we included stable isotopic data on potential viperfish predators to infer whether this species is being consumed by epipelagic and/or bathypelagic species. Based on data availability, local fauna, and literature information^10,12,35,36^, the following species were considered as potential predators and thus included in the analyses: *Ectreposebastes imus*, *Sphyraena barracuda*, *Coryphaena hippurus*, *Elagatis bipinnulata*, *Acanthocybium solandri*, *Katsuwonus pelamis*, and *Thunnus albacares*.

For GCA, each specimen had the stomach extracted and subsequently dissected under the stereoscope for content removal. Contents found in the mouth, oesophagus, and intestines were not considered in this study. Wherever possible, prey-size measurements to the nearest 0.1 mm were carried out with a binocular stereoscope using an ocular micrometric scale. Standard length for fishes; back of eye socket to tip of telson length (excluding terminal spines) for decapods; and tip of rostrum to tip of telson length (excluding terminal spines) for euphausiids were measured.

The vacuity index (VI, %) was calculated as follows: VI = Nv/Ne × 100, where Nv is the number of empty stomachs and Ne the total number of examined stomachs. Vacuity index was calculated for day, night, and both periods together. Dietary indexes for coupled stomachs were calculated to assess the importance of each prey item in viperfish diet: frequency of occurrence (%FO), numerical abundance (%N) and weight percentage (%W)^27^. Additionally, to estimate the niche breadth of viperfish, the Levin’s standardized index was calculated as follows: ${B}_{J}= \frac{1}{n-1}(\frac{1}{\sum {p}_{ij}^{2}}-1)$, where *B*_*j*_ is the Levin’s standardized index for the viperfish, whereas *p*_*i*_^*2*^_*j*_ is the proportion in weight of prey *i* in the diet of predator *j* and *n* is the number of prey categories. This index varies from 0 (species that feed on only one item) to 1 (species that feed on the same proportion of all evaluated items)^37^. Size-related differences were evaluated by comparing size classes through the non-parametric permutation procedure ANOSIM (Analysis of Similarity).

SIA were conducted on viperfish and its most probable prey and predator groups, including two fish larvae groups (Teleostei larvae 5–10 mm and Teleostei larvae 15–20 mm); five crustaceans; five gelatinous (divided into Siphonophorae and Thaliacea); eight zooplanktivorous fishes; and seven potential predators of viperfish (Table 2). Samples of Particulate Organic Matter (POM) were also included. For each fish and crustacean, white muscular tissue was extracted and cleaned with distilled water to remove exogenous material such as carapace, scales, and bones. Gelatinous organisms were used in whole. Entire zooplankton samples have been stored in Eppendorf micro tubes. Samples were dried in an oven at 60 °C for 48 h and grounded into a fine powder with a mortar and pestle. To obtain unbiased values of carbon stable isotope composition due to carbonates, zooplankton and POM samples were split in two subsamples. One zooplankton sub-sample was acidified by adding approximately 2 ml of 0.5 mol l^−1^ hydrochloric acid (HCl)^32,38^. POM sub-samples were exposed to hydrochloric acid (HCl) vapour. After 4 h, the filters and zooplankton were dried at 40 °C for 36 h. Untreated sub-samples of POM and zooplankton were analysed for nitrogen stable isotope composition and acidified one for carbon stable isotope composition. Each sample was analysed for carbon and nitrogen stable isotope ratios through a mass spectrometer (Thermo Delta V +) coupled to an element analyser (Thermo Flash 2000, interface Thermo ConFio IV) in the Platform Spectrometry Ocean (PSO, IUEM, France). SIA results for carbon (δ^13^C) and nitrogen (δ^15^N) were derived from the relation of the isotopic composition from the sample and a known standard: δ^13^C or δ^15^N = [(Rsample/Rstandard) – 1] × 10^3^; in which R corresponds to the ratio between ^13^C:^12^C or ^15^N:^14^N. As differential lipid contents can bias the interpretation of δ^13^C values, here we explored the potential lipid bias by using C:N ratios by mass and the relationship between C:N (i.e., lipid content) and δ^13^C. As samples were not treated to remove lipids before analysis to prevent loss of material, the few prey groups that exhibited C:N dynamics consistent with high lipid content (C:N > 3.5) were normalized using the equation for aquatic animals^31^: ∆δ^13^C = − 3.32 + 0.99 × C:N, where ∆δ^13^C is the change in δ^13^C caused by lipids and C:N is the carbon-to-nitrogen ratio (by mass) of the sample. To investigate the relationship between viperfish and potential prey and predators, isotopic values of carbon and nitrogen were analysed through a bi-dimensional plot. Further, viperfish trophic position (TP) was determined using the following formulae^39^:

$${\text{TP}} = \left( {{\updelta }^{15} {\text{N}}_{{{\text{consumer}}}} - {\updelta }^{15} {\text{N}}_{{{\text{baseline}}}} } \right)/{\text{TDF}} + {\text{ TP}}_{{{\text{baseline}}}}$$

where δ^15^N_consumer_ and δ^15^N_baseline_ are the δ^15^N values of the target consumer and the baseline respectively; TDF is the trophic discrimination factor and TP_baseline_ is the trophic position of the baseline. As POM may be influenced by the co-occurrence of detritus^40^ and microzooplankton in the water column^32^, primary consumers (TP2) are usually a better isotopic baseline to assess TP. Following the methodology of previous studies on the trophic position of mesopelagic, the baseline utilized was the zooplankton size fraction between 200–500 µm, which were mainly composed of herbivores copepods^32^ that act as primary consumers (TP2). To account for uncertainty associated with the index, a Bayesian model was incorporated in the calculation of TP using predict δ^15^N values of the viperfish and a TDF of 3.15‰ ± 1.28‰^41^. The R package *tRophicPosition*^42^ was run for isotopic trophic position calculations. To explore how trophic levels and carbon source might change across ontogenetic phases, the relationship between fish size and δ^13^C and δ^15^N were assessed through a least-squares linear regression analysis.

The bayesian mixing model, MixSIAR^43^, provide the most accurate estimations of source or prey contributions when tissue and species-specific discrimination factors are used^44^. Using the R package “*SIBER*”^45^, we applied mixing models to estimate the relative contribution of viperfish specific-prey utilization. To explore the relationships between source contribution and size, we performed three mixing models considering all size classes. Potential dietary endpoints applicable to viperfish included in mixing models were derived from stomach contents analyses, local fauna (e.g. the most abundant species of myctophids were selected), and published information^27,28,46^. The following prey species were included: i) *Euphausia gibboides* (Euphausiacea), ii) *Diaphus brachycephalus* (Myctophidae), iii) *Diaphus fragilis* (Myctophidae), iv) *Diaphus mollis* (Myctophidae), v) *Hygophum taaningi* (Myctophidae), vi) *Lampanyctus nobilis* (Myctophidae), vii) *Lepidophanes guentheri* (Myctophidae), viii) *Symbolophorus rufinus* (Myctophidae), ix) *Promethichthys prometheus* (Gempylidae).

## Results

### Oceanographic conditions

**Figure 2:**
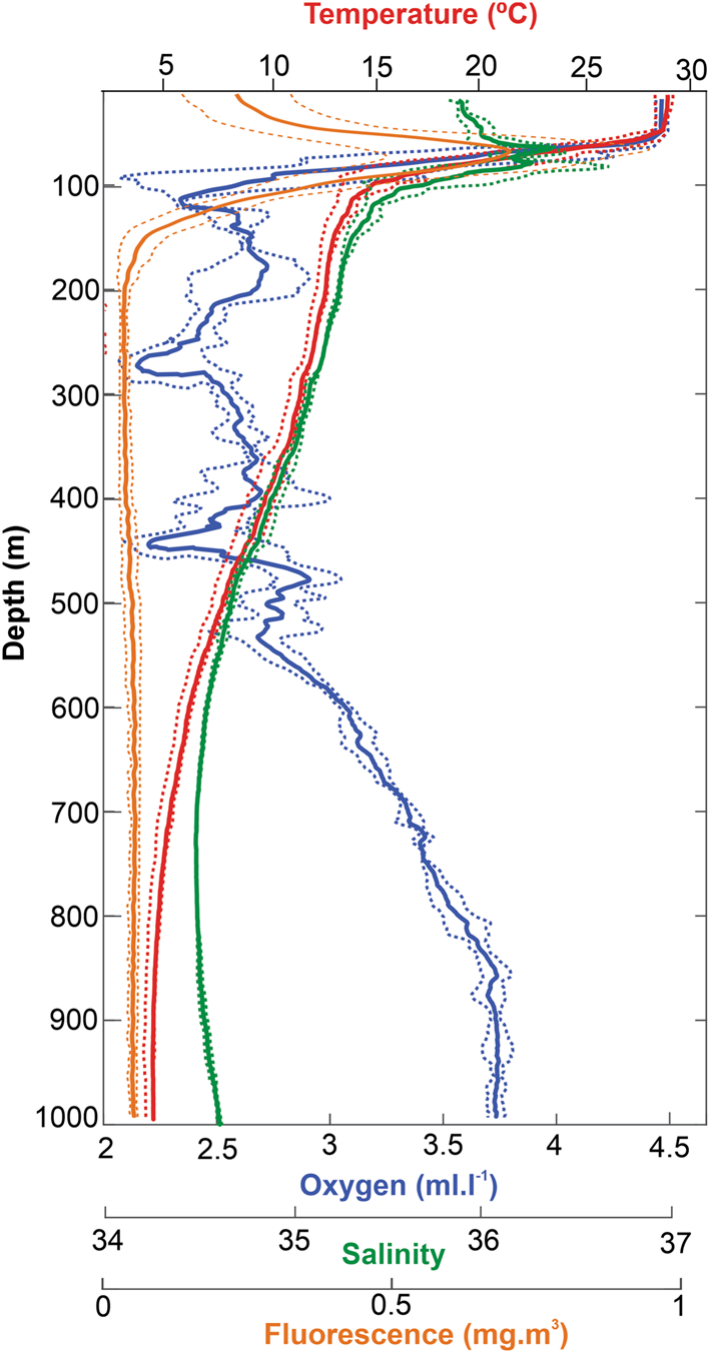
Mean and standard deviation of vertical profiles of temperature (red), dissolved oxygen (blue), salinity (green), and fluorescence (orange) in the study area during the survey.

Mean hydrological profiles (Fig. 2) revealed the presence of a surface mixed layer, characterized by warm waters (28 °C), extending down to ~ 50 m. Below, a sharp thermocline extended from the lower limit of the mixed layer to 130 m with a thermal difference of 12.3 °C. Vertical profile of salinity showed a layer of saline water within the thermocline, between 80 and 120 m. Dissolved oxygen concentration was homogeneous at the mixing layer, decreased at the upper limit of the thermocline with values less than 2.5 ml l^−1^ at ~ 100 m,  ~ 300 m, and ~ 450 m and then increased at depths higher than 550 m. The chlorophyll *a* fluorescence maximum was generally located at the upper limit of the thermocline.

### Vertical distribution, habitat, and migration

**Figure 3:**
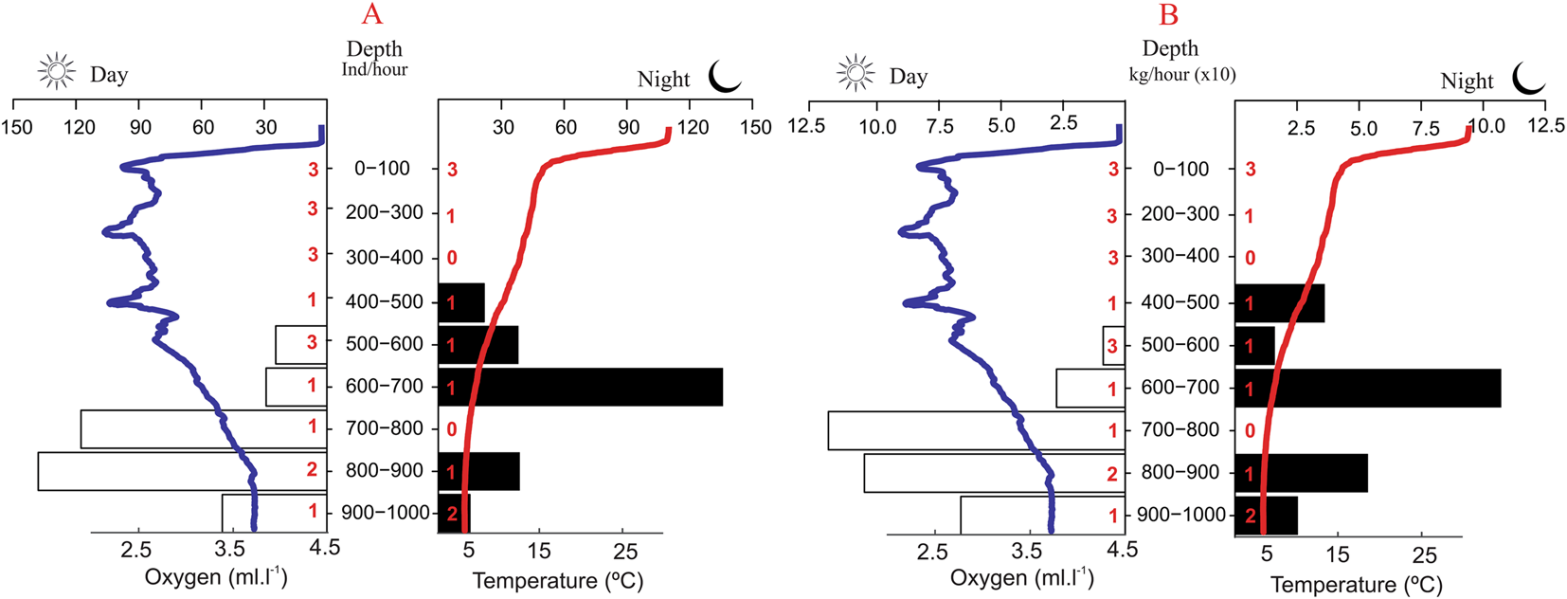
Average relative abundance in individuals.hour^−1^ (A) and kilogram.hour^−1^ (B) per depth strata and day period of the viperfish *Chauliodus sloani*. Coloured lines represent the average vertical profile of dissolved oxygen (blue) and temperature (red) for both day and night times. Red numbers represent the number of trawls per depth strata and period of the day.

**Figure 4:**
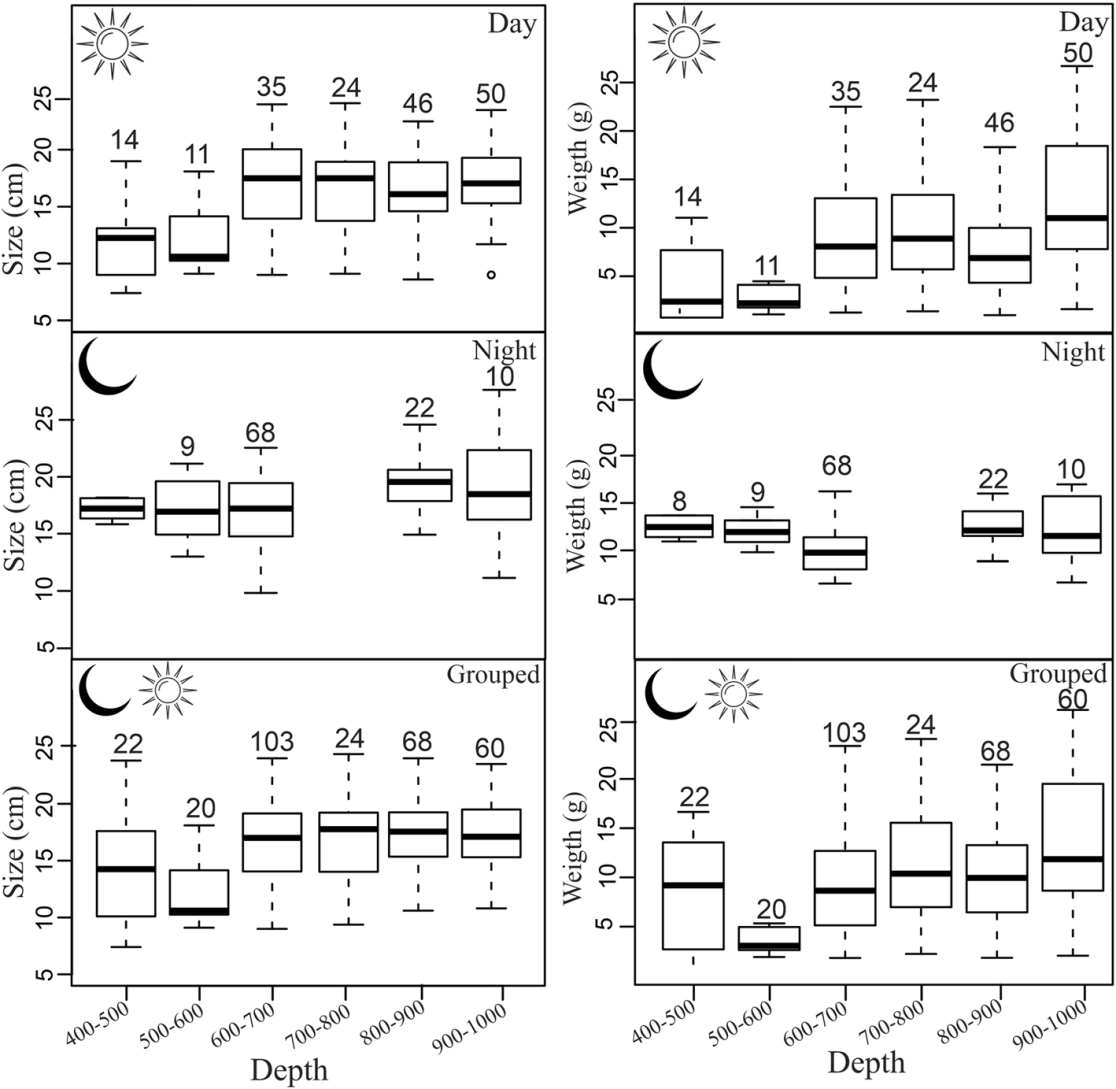
Boxplot of standard length and total weight per size classes and period of the day for the viperfish *Chauliodus sloani*. The depth layer 700–800 m was not sampled at night. Black horizontal lines and boxes represent median values and interquartile ranges, respectively. Dashed lines represent the data range limits. Numbers above the boxes represent the quantity of specimens per depth strata.

A total of 304 specimens of viperfish was collected and utilized to investigate vertical habitat and behaviour. The mean and standard deviation of the relative index of abundance were 62.3 ± 87.2 ind.hour^−1^ (0.62 ± 0.86 kg.hour^−1^), ranging from 2.6 ind.hour^−1^ (0.03 kg.hour^−1^) to 340 ind.hour^−1^ (3.37 kg.hour^−1^). Vertically, viperfish were captured only between 400 to 1000 m, showing abundance peaks at 700–900 m (daytime) and 600–700 m (night-time). Both day and night specimens were found between 400 and 1000 m (Fig. 3), suggesting that only part of the population performs diel vertical migration. Additionally, size and weight varied significantly (p = 0.02) with the diel period and depth strata, indicating a possible ontogenetic shift on distribution and vertical migration pattern. At daytime, size distribution was heterogeneous among depth layers with larger organisms distributed below 500 m (difference of ± 5 cm/5 g). At night, however, larger individuals seem to migrate upwards, resulting in a more homogeneous size distribution (difference of ± 1 cm/2 g) according to depth layers. Coupling both periods, larger and heavier individuals were found at depths below 500 m (Fig. 4, Suppl. Material 3). *Chauliodus sloani* was captured in temperature ranging from 5 to 12 °C, well below the thermocline zone. Considering dissolved oxygen, the species was caught between 2.5 ml l^−1^ and 3.8 ml l^−1^ (Fig. 3).

### Trophic ecology

One hundred and ninety-seven individuals (7–25 cm SL) were dissected for investigation on the viperfish carbon source through GCA. From that, 76 (39%) had stomachs with content and were utilized for further analyses. The vacuity index was 72% for daytime, 50% for night, and 61% for pooled periods. Considering all size classes, *C. sloani* feds largely on myctophids of the genus *Diaphus* (23% by weight, noted hereafter 23%W; 10–30 mm SL) and unidentified myctophids (36%W; 20–36 mm). Unidentified Teleostei (which may also include myctophids) was likewise important (31%W; 11–38 mm), followed by a few specimens of *Hygophum* sp. (3.4%W), *C. sloani* (2.4%W; 38 mm), *Cyclotone* spp. (1.5%W; 27 mm), Gempylidae (0.3%W; 35 mm) and Euphausiidae (0.2%W; 26 mm) (Table 1). No crustaceans were found in stomachs of individuals larger than 15 cm. The low value of Levins standardized index (< 15 cm: 0.22; > 15 cm: 0.30; Pooled Sizes: 0.17) indicated a restricted niche breadth for all size classes, highlighting the strong piscivorous habit of this species. Overall, lager individuals presented a higher niche breadth.

**Table 1:** Diet composition of viperfish *Chauliodus sloani* utilized in gut content analyses and dietary indexes calculated for each prey item: abundance percentage (%N), weight percentage (%W), frequency of occurrence (%F), number of specimens analysed (N), number of stomachs with content (NSC), vacuity index (%VI), vacuity index day (%VD), vacuity index night (%VN) and niche breadth (*B*_*j*_)*.*

Prey item		Grouped Sizes			Size class: 7–15 cm			Size class: 15–25 cm		
		N:197; NSC:76; *B*_*j*_:0.17			N:55; NSC:16; *B*_*j*_:0.22%			N:142; NSC:60; *B*_*j*_:0.30		
		%VI:61; %VD: 72; %VN:50			VI:71; %VD78; %VN:58			%VI:58; %VD:68; %VN:49		
Group	Taxa	%FO	%N	%W	%FO	%N	%W	%FO	%N	%W
Crustaceans	Euphausidae	1.3	3.1	0.2	7.1	11.1	2.51	-	-	-
	Decapoda	1.3	0	0.1	7.1	11.1	1.7	-	-	-
Fish	*Chauliodus sloani*	1.3	3.1	2.4	7.1	11.1	29.3	-	-	-
	*Cyclotone* spp.	1.3	3.1	1.5	-	-	-	2.7	4.3	2.1
	Gempylidae	1.3	3.1	0.7	-	-	-	2.7	4.3	1.0
	*Diaphus* sp.	2.6	6.2	23.4	-	-	-	5.4	8.7	33.2
	*Hygophum* sp.	1.3	3.1	3.4	-	-	-	2.7	4.3	4.8
	Myctophidae	15.7	28.1	36.2	7.1	11.1	1.5	24.0	39.1	33.2
	Unidentified Teleostei	39.4	50.0	31.8	71.4	55.5	64.7	49.0	39.1	25.5

**Figure 5:**
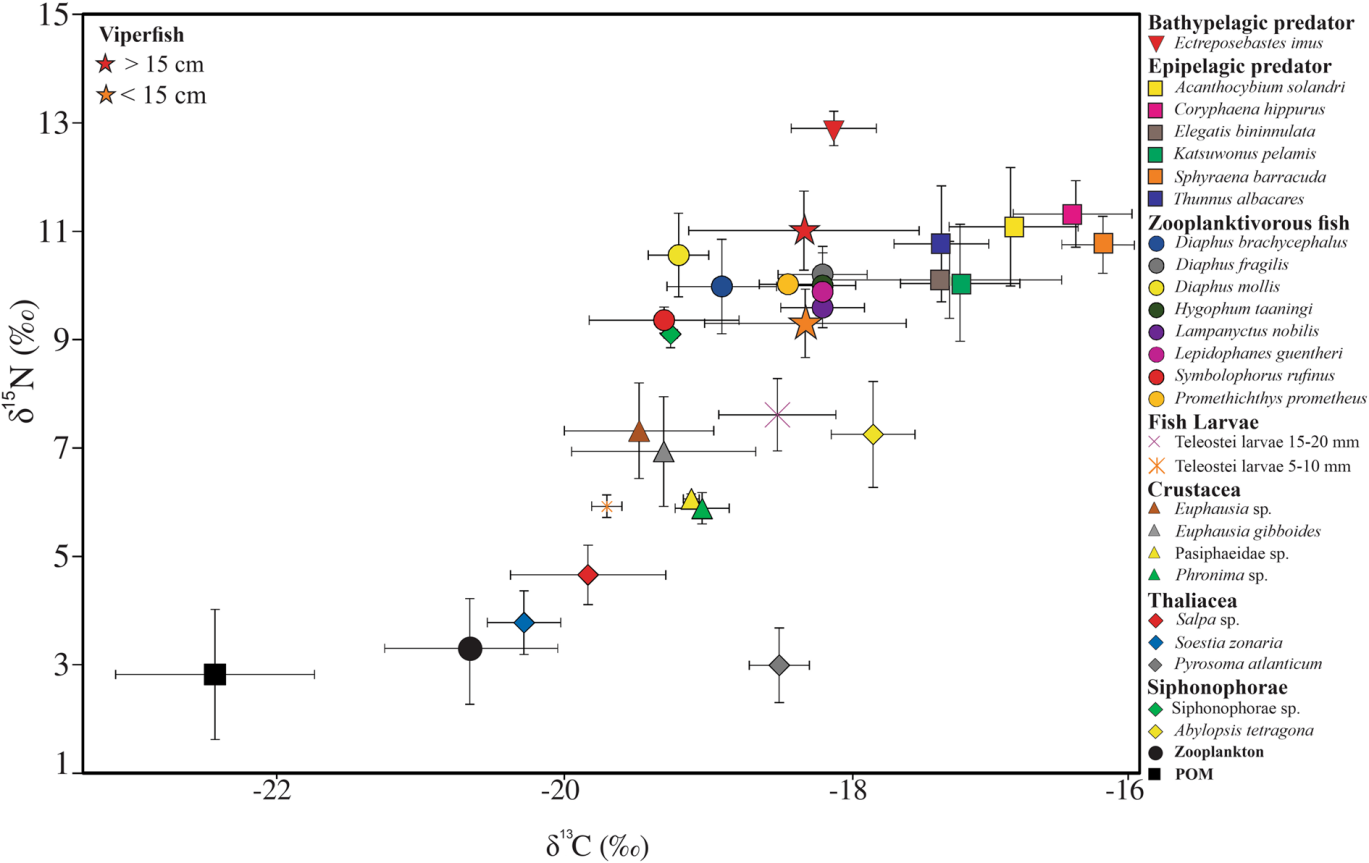
Stable carbon and nitrogen isotope values of particulate organic matter (POM), the viperfish *Chauliodus sloani* and its potential predators, potential preys, and lower trophic levels.

**Figure 6:**
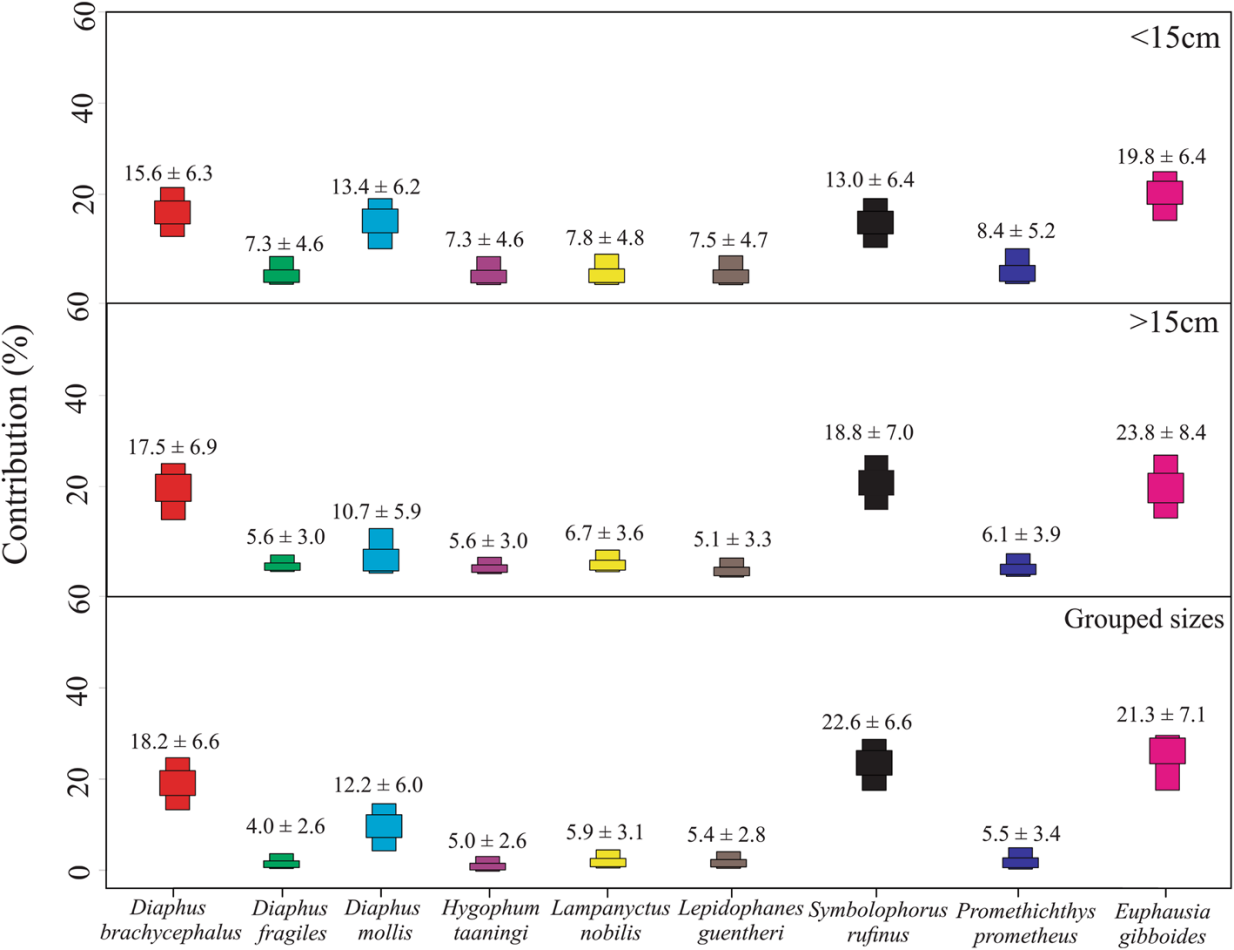
Estimated contribution in % (numbers; mean ± SD) based on stable isotope mixing model of potential prey to the diet of the viperfish *Chauliodus sloani*. Coloured boxes represent 25% and 50% quantiles.

Considering stable isotope analyses, 26 taxa were utilized to assess viperfish trophic ecology (Table 2). Overall, the mixing models and biplot analyses were consistent with GCA, suggesting a tight trophic interaction with fishes, especially myctophids (e.g. *Diaphus brachycephalus* and *Symbolophorus rufinus*) (Figs. 5 and 6). Moreover, the mixing model for all size classes revealed a higher isotopic contribution of euphausiids that could not be observed in GCA (Fig. 5). Mean δ^15^N values (< 15 cm = 9.3 ± 0.6‰; > 15 cm = 11.1 ± 0.7‰) and trophic levels (TPsia: < 15 cm = 3.9 ± 0.1; > 15 cm = 4.3 ± 0.1; grouped = 4.1 ± 0.11) were significantly different among ontogenetic phases. Considering δ^13^C values (< 15 cm = − 18.3 ± 0.2‰; > 15 cm = − 18.3 ± 0.1‰), no significant differences were observed among ontogenetic phases (Suppl. Material 2; Table 2; p < 0.05). The consistency in carbon and nitrogen values between the viperfish and the bathypelagic predator *Ectreposebastes imus* indicate a likely tight trophic linkage between them. The difference in δ^13^C isotopic values between the viperfish and epipelagic predators, however, does not indicate that viperfish could significantly contribute to their feeding regime.

**Table 2:** Number of samples, standard length, and isotopes values of the viperfish *Chauliodus sloani* and its potential predators (Bat.pred–bathypelagic predator; Epi.pred–epipelagic predator), potential prey, and lower trophic levels (LTL). *Species corrected for lipid.

Group	Species	Category	N	Standard length (cm)	δ^13^C (‰)	δ^15^N (‰)	C:N
				Mean ± SD	Mean ± SD	Mean ± SD	Mean ± SD
Stomiidae	*Chauliodus sloani* (> 15 cm)	–	10	18.1 ± 1.3	− 18.3 ± 0.1	11.1 ± 0.7	3.3 ± 0.1
	*Chauliodus sloani* (< 15 cm)	–	17	13.6 ± 1.5	− 18.3 ± 0.2	9.3 ± 0.6	3.3 ± 0.1
Setarchidae	*Ectreposebastes imus*	Bat.pred	5	19.1 ± 1.7	− 19.1 ± 0.3	12.8 ± 0.2	4.3 ± 0.2
Sphyraenidae	*Sphyraena barracuda*	Epi.pred	7	151.2 ± 30.0	− 16.2 ± 0.4	10.7 ± 0.5	3.2 ± 0.1
Coryphaenidae	*Coryphaena hippurus*	Epi.pred	6	85.2 ± 12.0	− 16.4 ± 0.4	11.3 ± 0.6	3.2 ± 0.1
Carangidae	*Elagatis bipinnulata*	Epi.pred	6	53.3 ± 10.4	− 19.3 ± 0.2	9.3 ± 0.5	3.4 ± 0.2
Scombridae	*Acanthocybium solandri*	Epi.pred	8	100.0 ± 35.0	− 16.8 ± 0.4	11.0 ± 1.0	3.2 ± 0.1
	*Katsuwonus pelamis*	Epi.pred	3	44.6 ± 4.1	− 17.2 ± 0.4	10.2 ± 1.0	3.2 ± 0.1
	*Thunnus albacares*	Epi.pred	12	65.0 ± 20.0	− 17.3 ± 0.2	10.7 ± 1.0	3.1 ± 0.1
Myctophidae	*Diaphus brachycephalus*	prey	10	5.0 ± 2.1	− 18.9 ± 0.3	9.9 ± 0.8	3.4 ± 0.1
	*Diaphus fragilis*	prey	11	7.3 ± 0.4	− 18.2 ± 0.3	10.2 ± 0.5	3.4 ± 0.1
	*Diaphus mollis*	prey	5	5.2 ± 0.3	− 19.2 ± 0.2	10.5 ± 0.7	3.4 ± 0.1
	*Hygophum taaningi*	prey	9	5.5 ± 0.2	− 18.2 ± 0.2	10.0 ± 0.6	3.3 ± 0.1
	*Lampanyctus nobilis*	prey	7	7.4 ± 1.5	− 18.2 ± 0.2	9.5 ± 0.3	3.3 ± 0.1
	*Lepidophanes guentheri*	prey	13	5.7 ± 0.6	− 18.2 ± 0.2	9.8 ± 0.7	3.3 ± 0.1
	*Symbolophorus rufinus*	prey	6	5.7 ± 0.3	− 19.3 ± 0.2	9.3 ± 0.5	3.4 ± 0.1
Gempylidae	*Promethichthys prometheus*	prey	3	14.2 ± 2.0	− 18.4 ± 0.2	10.0 ± 0.1	3.3 ± 0.1
Fish larvae	Teleostei larvae 15–20 mm	prey	6	–	− 18.5 ± 0.4	7.1 ± 0.6	3.2 ± 0.1
	Teleostei larvae 5–10 mm	prey	10	–	− 19.6 ± 0.1	5.9 ± 0.2	3.2 ± 0.1
Crustacea	*Euphausia gibboides*	prey	6	1.5 ± 0.1	− 19.3 ± 1.0	6.9 ± 0.2	3.2 ± 0.1
	*Euphausia* sp.	prey	3	1.4 ± 0.1	− 19.4 ± 0.5	7.3 ± 0.8	3.2 ± 0.1
	Pasiphaeidae sp.	prey	3	–	− 19.1 ± 0.0	6.0 ± 0.1	3.1 ± 0.1
	*Phronima* sp.	prey	3	–	− 19.0 ± 0.1	5.8 ± 0.1	3.6 ± 0.2
Siphonophorae	*Abylopsis tetragona*	LTL	3	–	− 17.8 ± 0.2	7.2 ± 1.0	3.3 ± 0.1
	Siphonophorae sp.	LTL	3	–	− 19.2 ± 0.0	9.1 ± 0.2	3.4 ± 0.1
Thaliacea	*Salpa* sp.*	LTL	6	–	− 19.8 ± 0.5	5.4 ± 0.1	4.5 ± 0.7
	*Soestia zonaria*	LTL	6	–	− 20.2 ± 0.2	3.7 ± 0.5	3.3 ± 0.1
	Pyrosoma atlanticum*	LTL	11	–	− 18.5 ± 0.2	2.9 ± 0.6	5.4 ± 0.2
Zooplankton		LTL	19	–	− 19.4 ± 0.3	3.0 ± 0.6	4.5 ± 0.5
POM		LTL	17	–	− 22.4 ± 0.6	2.8 ± 1.2	–

## Discussion

Here we analysed the habitat, vertical migration, and trophic ecology of the viperfish *Chauliodus sloani* to further understand the ecology and thus functional role of mesopelagic micronektivores. Among others, we combine our results with previous studies and examine through a conceptual model how latitudinal change in physicochemical conditions can modulate the viperfish’s behaviour. For instance, we show that physical drivers are regulating both patterns of movements and trophic interactions of this species, with possible consequences for ecological processes as energy transfer among vertical oceanic layers. Moreover, we address some of the potential contribution of this species to the oceanic carbon storage. Finally, for the first time we describe the ecology of a mesopelagic micronektivore along the western Tropical Atlantic (WTA), providing further information on an important and poorly known deep-sea species.

### Methodological constrains

Some considerations should be made before the interpretation of our results. First, although we took precautions to avoid the collection of specimens during the lowering or hoisting of the net (see methodology), our gear did not have an opening or closing mechanism, allowing the collection of some species during these processes. Moreover, our samples were focused on mesopelagic waters and distribution patterns at layers deeper than 1000 m could not be assessed. Therefore, here we focused on major patterns of vertical behaviour on epipelagic and mesopelagic waters (0–1000 m depth), avoiding precise delimitations of vertical distribution and standing stock calculations. Second, diet determination from isotopic mixing models is closely related to the trophic discrimination factor (TDF) and sources utilized to run the analysis^47^. Hence, despite we carefully selected TDF values and prey groups (see methodology), the inclusion of different prey may provide further insights on the viperfish’s trophodynamics^47,48^. Overall, the results presented here are not intended to exhaustively describe the ecological aspects of the viperfish. Instead, they increase the understanding of an important and understudied species, as well as provide novel insights on several aspects of its ecology.

### Vertical distribution, habitat, and migration

Based on our data, in the WTA, the viperfish is the most important mesopelagic species in terms of biomass and fifth more abundant (4% of the total; L. N. Eduardo, unpublished data). Indeed, *Chauliodus sloani* represented 13% of the total biomass collected, followed by *Borostomias* sp. (10%), *Sternoptyx diaphana* (5%), *Melamphaes polylepis* (5%), and *Argyropelecus affinis* (4%)(L. N. Eduardo, unpublished data). The viperfish inhabits depth layers below 400 m, i.e. at temperatures lower than 12 °C and oxygen levels between 2.3 and 3.7 ml l^−1^. In mesopelagic waters, the abundance of this species peaked at 700–900 m at daytime and 600–700 m at night, indicating a pattern of restricted vertical migration where part of the population seems to migrate upwards at night. Moreover, we evidenced ontogenetic spatial variations (e.g. larger and heavier individuals distributed deeper, below 500 m) and asynchronous patterns of migration, where the entire population does not respond synchronously to diel variation in light intensity (segregating by depth and/or size).

This vertical ascension and size segregation have been previously reported in sub-tropical and temperate zones (Table 3). Interestingly, at all these locations, viperfish has been recorded in epipelagic waters, which was not the case in our data. Oxygen levels and temperature are two oceanic features known to constrict the vertical distribution of mesopelagic fish species^5,49,50^. The viperfish is known to occupy suboxic waters (e.g. 1.0 ml l^−1^)^27^, seemingly to support much lower oxygen levels than those reported here. Therefore, vertical distribution differences among oceanic regions may be caused by the warmer epipelagic waters of tropical regions that may be preventing the ascension of this species up to shallow layers. Indeed, by coupling our data with previously information we observe that, independently of the depth, the upper thermal limit of the viperfish ranges from 12° to 15 °C (Table 3). Hence, it is likely that temperature may be shaping the migration patterns of this species. While viperfish ascend to epipelagic waters in sub-tropical and temperate regions, in tropical areas it seems to remain at greater depths. One exception is the record of this species in the superficial tropical waters of Somalia^27^. However, this region is affected by seasonal monsoon conditions and has a strong upwelling, which leads to the cooling of epipelagic waters^51^. This exception reinforces our hypothesis that temperature may be ruling the epipelagic rise of the viperfish.

**Table 3:** List of previous records of the viperfish *Chauliodus sloani*, including the location of occurrence, climatic zone, epipelagic record, depth, and temperature range.

Location	Climatic zone	Epipelagic record	Depth range (m)	Temperature range (°C)	References
Western Tropical Atlantic	Tropical	No	400–1000	5–12	This study
South Pacific (Tasmania)	Temperate	Yes	100–900	5–13	^74–76^
Northeastern Atlantic	Temperate	Yes	100–600	10–12	^77,78^
Eastern Gulf of Mexico	Subtropical	Yes	150–800	4–15	^23^
Southwestern Indian Ocean	Subtropical	Yes	100–700	4–15	^79,80^
Arabian Sea (Somalia)	Tropical	Yes	100–1500	5–15	^27^
Mid-Atlantic Ridge	Temperate	Yes	50–2900	6–12	^81–83^

### Trophic ecology

Differences on the vertical distribution along tropical and temperate regions seems also to reflect in the trophic links of the viperfish. While *C. sloani* represents one of the most important prey items of epipelagic predators in several locations^10,35,36,52^, previous studies addressing the trophic ecology of epipelagic predators along the WTA do not mention a trophic relationship with the viperfish^9,53^. Moreover, SIA results do not evidence a well-defined trophic relationship between the viperfish and potential epipelagic predators. It might reflect the low probabilities of predator–prey encounters, as viperfish and epipelagic predators may not be sharing the same vertical space. On the contrary, the isotopic compositions of the viperfish and the bathypelagic predator *Ectreposebastes imus* are well-matched. The trophic link between bathypelagic predator and the viperfish has been also noted worldwide^54–56^.

Based on its prey, the viperfish is a predator with a restricted niche breadth that heavily feeds on zooplanktivorous fishes, especially myctophids (at least 50% of prey items). This is supported by the mixing models, which show a potentially high contribution of Myctophidae, especially *Diaphus brachycephalus* and *Symbolophorus rufinus* (Fig. 6). This high contribution of myctophids has been also reported in the Central Mediterranean Sea^28^, Pacific Ocean^57^, Arabian Sea^27^, North Atlantic Ocean^58^, and Indian Ocean^59^. Euphausiids were also found as a prey item, both here and in previous studies^57^, but in a lesser extension. Larger individuals (> 15 cm; TP_sia_: 4.3) fed on larger prey and were more enriched in ^15^N than small specimens (< 15 cm; TP_sia_: 3.9), reflecting possible ontogenetic trophic shifts and differences on the prey-size consumption.

Overall, considering previous studies and our data, we conclude that myctophids are the most important prey item of the viperfish, followed by few other Teleostei species (e.g. Gempylidae sp., *Cyclotone* spp.), and euphausiids. Following the diel vertical behaviour of zooplankton, most myctophids (including main viperfish prey, e.g. *D. brachycephalus* and *Hygophum* spp*.*) forage in epipelagic zones at night and vertically migrate and form high-density biological layers in deeper waters in search of predator refuge during daytime^12,60–62^. Indeed, species of Myctophidae are amongst the most important epipelagic zooplankton consumers, feeding up to 30% of their daily stocks^61,62^. Likewise, most of the euphausiids species undergo diel vertical migrations, where they move upwards at night, usually in the layer of maximum chlorophyll concentration, seeking a high density of prey^63,64^. We thus deduce that most viperfish prey are epipelagic migrants that forage on surface waters.

### Potential contribution for the Biological Carbon Pump

The Biological Carbon Pump (BCP) is the active and passive transport of particulate organic carbon produced in the ocean surface by photosynthesis to the deep ocean^7,8^. Given their behaviour, high biomass and feeding ecology, mesopelagic micronektivores potentially contribute to the active part of this process^8,65–67^. Indeed, they may be isolated from epipelagic predators and they are directly and/or indirectly (through their prey) connected to epipelagic waters where photosynthetic processes occur^5,23,25,68^. Carbon storage depends on the depth difference between the ingestion of carbon and its release by respiration, excretion, defecation, and mortality^8,62,65,67,69^. For instance, carbon may be sequestered for longer than a year when released at mesopelagic waters, and for up to centuries when egested on deeper-water masses (generally greater than 1,000 m)^6,7^. Conversely, carbon may not be stored when vertical migrants are consumed by epipelagic predators and/or released at surface waters^6,7,70^. Hence, the contribution of mesopelagic micronektivores to the BCP depends on their diel vertical migration as well as the one of their prey and predators^70,71^.

Based on our data, the viperfish is the most abundant mesopelagic micronektivore in the WTA. This species remains at deep waters full-time, is away from epipelagic predators, and heavily preys on migrant myctophids, which otherwise would return and release carbon in epipelagic waters. Additionally, at epipelagic waters myctophids are extensively preyed by epipelagic predators. Therefore, this species likely contributes to carbon storage, once it supports the storage of organic matter actively vertically transported through their prey. Moreover, viperfish are preyed by higher trophic levels (e.g. *Ectreposebastes imus*) that perform diel migrations from bathypelagic depth to feed at the lower mesopelagic zone (500–1000 m). This relationship may also accelerates carbon sequestration into the deep-sea. However, the BCP is a complex process^67,69,72^ and here we focused only on ecological drivers (vertical behaviour and trophodynamics) that could enhance this activity. Further studies are required to thoroughly investigate the contribution of mesopelagic micronektivores on the BCP. For instance, future investigation should measure and/or estimate the carbon flux of these species through respiration, gut flux, excretion, and mortality^8,65,67,69^. Additionally, to properly understand the extension of this process, estimated carbon fluxes must be contrasted with the gravitational flux of particulate organic matter.

### Conceptual model

**Figure 7:**
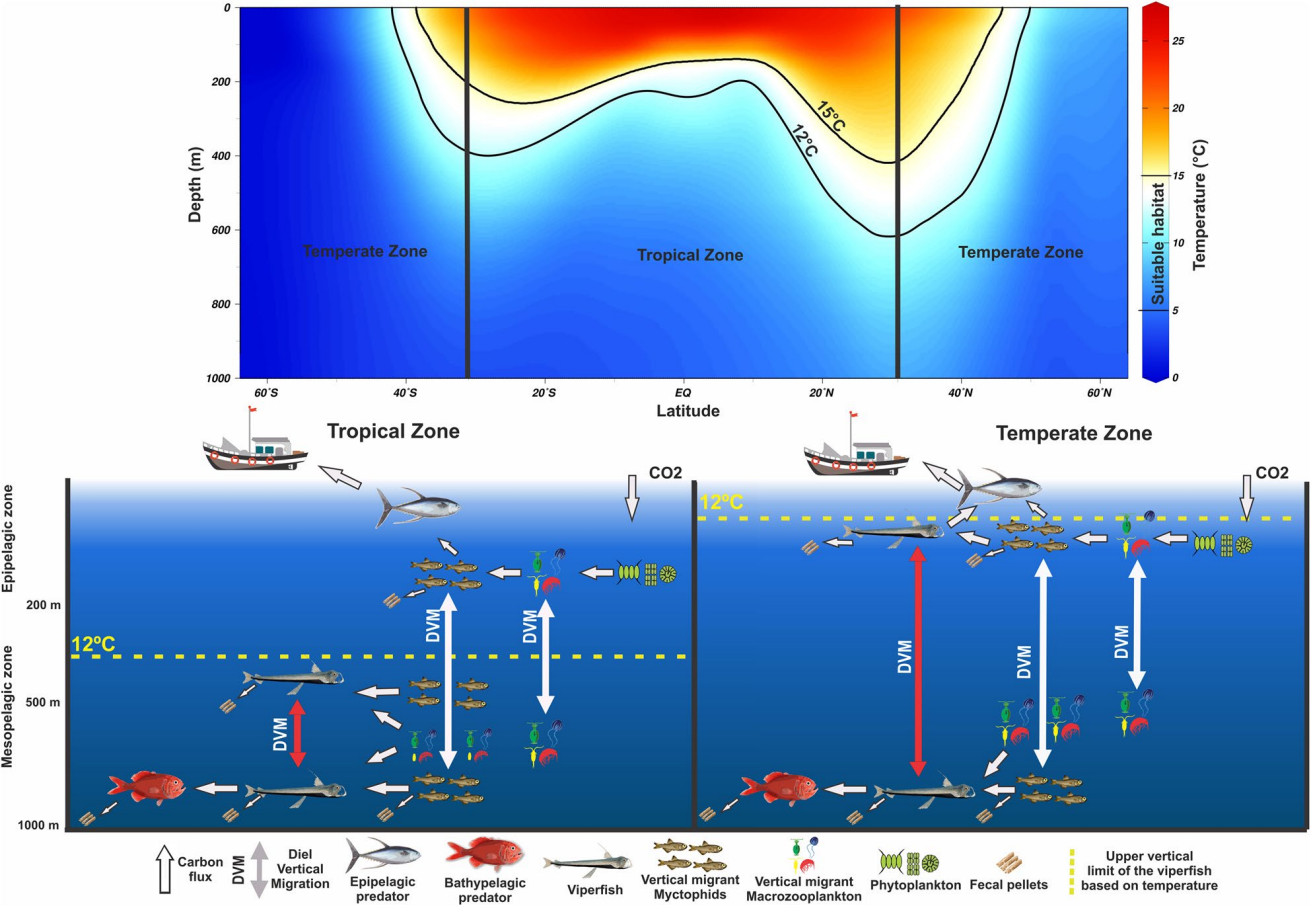
Conceptual model exhibiting global suitable vertical habitat of the viperfish *Chauliodus sloani* based on temperature profiles (Source: Word Ocean Atlas^73^) and differences in the vertical migration and trophic interactions of this species in the tropical and temperate waters. Temperature information from the upper panel refers to the meridional Sect. 30°.

By combining our results with previous works, we constructed a conceptual model explaining how temperature might influence both trophic ecology and vertical movements of the viperfish (Fig. 7). We observed that temperature (12–15 °C) is likely restricting its upper limit of distribution and thus affecting its vertical habitat and trophodynamics. For instance, in the WTA, and probably most of tropical waters, the viperfish likely stay full-time breathing, excreting, and serving as prey (e.g. for bathypelagic predators) at deep layers (below 400 m). In most temperate regions, however, they ascend to superficial waters where they are consumed by epipelagic predators and release carbon where its remineralization is the greatest (0–200 m). More broadly, based on the viperfish case, we show that the ecology and thus potential contribution of micronektivores to the carbon storage is expected to vary geographically, modulated by the latitudinal change in sea temperature.

## Conclusion

Here we combined novel information on the viperfish trophodynamics and migratory behaviour in relation to physicochemical conditions (oxygen and temperature) to further understand the ecology and thus functional role of mesopelagic micronektivores. We demonstrate that, in the western Tropical Atlantic, the viperfish is amongst the most important mesopelagic micronektivore in terms of abundance and biomass. This species remains full-time at deep waters, heavily preys on myctophids, and presents spatial and trophic ontogenetic shifts. Temperature restricts its vertical distribution. Therefore, its ecology and functional roles are expected to be modulated by the latitudinal change in temperature. Moreover, we address some of its potential contribution to carbon storage and suggest further research.

Our findings indicate that the ecology and thus functional role of mesopelagic micronektivores may be more complex than previously thought, providing new perspectives on their trophic ecology, habitat, and migratory behaviour. With the predicted and observed effects of climatic change^16,17^, pollution^18^, and exploitation of deep-sea resources, we reaffirm that the structure and function of deep-sea ecosystems could undergo changes that, given the current state of knowledge, may go mostly unnoticed by scientists, marine resource managers, and conservation biologists. Studying the variability of biological behaviors of mesopelagic fishes is critical to further understand their ecology, conservation, and thus several ecosystem processes.

## Supplementary information

Supplementary Information.
