# Supplementary material for: Trophic ecology, habitat, and migratory behaviour of the viperfish Chauliodus sloani reveal a key mesopelagic player
Source: Sci Rep. 2020 Dec 2;10:20996. doi: 10.1038/s41598-020-77222-8 (PMC7710699; doi:10.1038/s41598-020-77222-8)
Supplement: Supplementary file 1 — Supplementary Information. [file 41598_2020_77222_MOESM1_ESM.docx]

**Trophic ecology, habitat, and migratory behaviour of the viperfish *Chauliodus sloani* reveal a key mesopelagic player**

Leandro Nolé Eduardo^1,2*^, Flávia Lucena-Frédou^1^, Michael Maia Mincarone^3^, Andrey Soares^1^, François Le Loc’h^4^, Thierry Frédou^1^, Frédéric Ménard^5^, Arnaud Bertrand^1,2,6^

^1^ Universidade Federal Rural de Pernambuco, Departamento de Pesca e Aquicultura, Recife, PE, Brazil.

^2^ Institut de Recherche pour le Développement (IRD), MARBEC, Univ. Montpellier, CNRS, Ifremer, IRD, Sète, France.

^3^ Universidade Federal do Rio de Janeiro, Instituto de Biodiversidade e Sustentabilidade, Caixa Postal 119331, Macaé, RJ, 27910-970, Brazil.

^4^ IRD, Univ. Brest, CNRS, Ifremer, LEMAR, IUEM, F-29280 Plouzane, France.

^5^ Aix Marseille Univ., Université de Toulon, CNRS, IRD, MIO, UM110, Marseille, France.

^6^ Universidade Federal de Pernambuco, Departamento de Oceanografia, Recife, PE, Brazil.

**SUPPLEMENTARY MATERIAL**

Supplementary Material 1 – Number of trawls per depth strata and period of the day.

| Depth Strata | Day | Night |
| --- | --- | --- |
|  |  |  |
| 10-100 | 3 | 3 |
| 100-200 | 3 | 1 |
| 200-300 | 3 | - |
| 300-400 | 1 | 1 |
| 400-500 | 3 | 1 |
| 500-600 | 1 | 1 |
| 600-700 | 1 | 1 |
| 700-800 | 2 | - |
| 800-900 | 1 | 1 |
| 900-1000 | 2 | 2 |


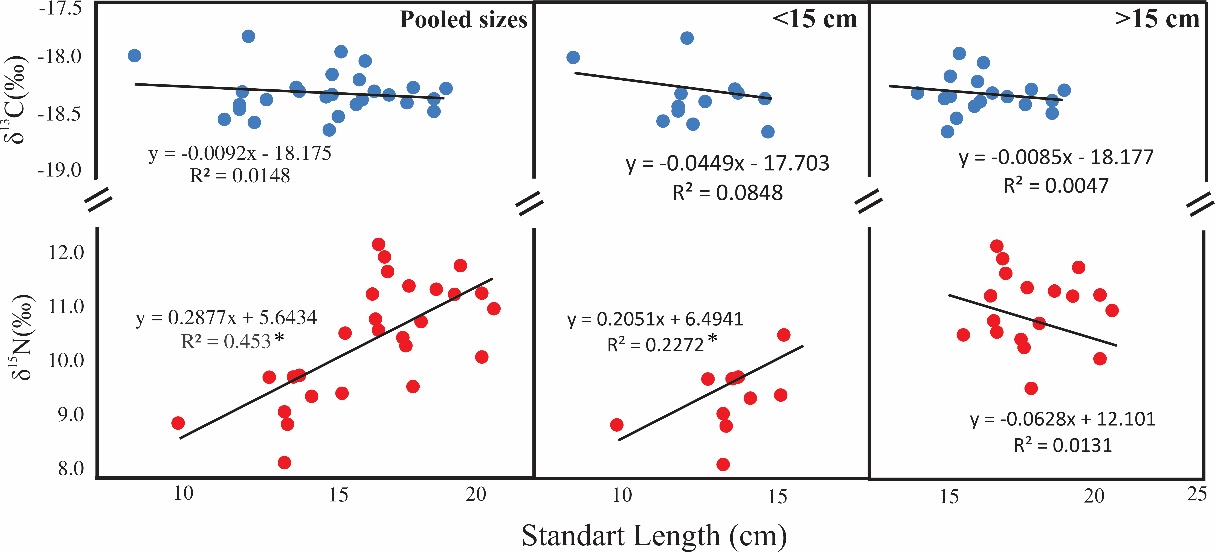


Supplementary Material 2 – Results of least-squares regression analysis between standard length (cm) and δ^15^N and δ^13^C values of the viperfish *Chauliodus sloani.** relationships presenting significant statistical differences (p <0.05).


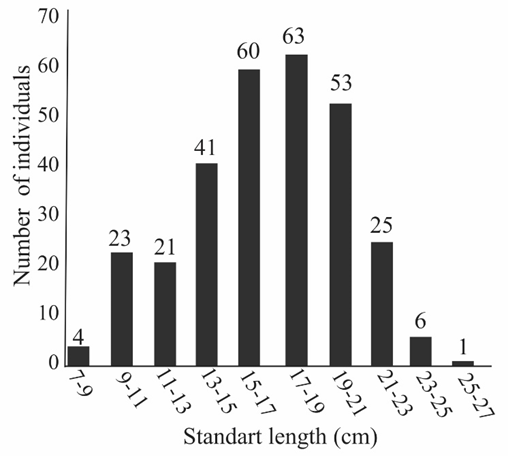


Supplementary Material 3– Histogram of the number of individuals per length. Numbers above bars represent the number of individuals in each size class.
